# DNA Methyltransferase 3B Gene Promoter and Interleukin-1 Receptor Antagonist Polymorphisms in Childhood Immune Thrombocytopenia

**DOI:** 10.1155/2012/352059

**Published:** 2012-09-19

**Authors:** Margarita Pesmatzoglou, Marilena Lourou, George N. Goulielmos, Eftichia Stiakaki

**Affiliations:** ^1^Department of Pediatric Hematology-Oncology, University of Crete, University Hospital of Heraklion, 71110 Heraklion, Crete, Greece; ^2^Laboratory of Molecular Medicine and Human Genetics, Department of Medicine, University of Crete, 71003 Heraklion, Crete, Greece

## Abstract

Primary immune thrombocytopenia (ITP) is one of the most common blood diseases as well as the commonest acquired bleeding disorder in childhood. Although the etiology of ITP is unclear, in the pathogenesis of the disease, both environmental and genetic factors including polymorphisms of TNF-a, IL-10, and IL-4 genes have been suggested to be involved. In this study, we investigated the rs2424913 single-nucleotide polymorphism (SNP) (C46359T) in DNA methyltransferase 3B (*DNMT3B*) gene promoter and the VNTR polymorphism of IL-1 receptor antagonist (*IL-1 Ra*) intron-2 in 32 children (17 boys) with the diagnosis of ITP and 64 healthy individuals. No significant differences were found in the genotype distribution of *DNMT3B* polymorphism between the children with ITP and the control group, whereas the frequency of allele T appeared significantly increased in children with ITP (*P* = 0.03, OR = 2, 95% CI: 1.06–3.94). In case of *IL-1 Ra* polymorphism, children with ITP had a significantly higher frequency of genotype I/II, compared to control group (*P* = 0.043, OR = 2.60, 95% CI: 1.02–6.50). Moreover, genotype I/I as well as allele I was overrepresented in the control group, suggesting that allele I may have a decreased risk for development of ITP. Our findings suggest that rs2424913 *DNMT3B* SNP as well as *IL-1 Ra* VNTR polymorphism may contribute to the susceptibility to ITP.

## 1. Introduction

Primary immune thrombocytopenia, commonly referred to as idiopathic thrombocytopenic purpura (ITP), is one of the most common blood diseases as well as the commonest acquired bleeding disorder in childhood. The affected children are young and previously healthy, and they typically present with a sudden onset of petechiae or purpura 2-3 weeks after a viral infection or immunization. Complete remission occurs in at least 2/3 of cases within 6 months of initial diagnosis [1, 2]. ITP is pathophysiologically characterized by a low circulating platelet count due to the production of autoantibodies against platelet glycoproteins, especially against GPII_b_/III_a_ and I_b_/IX, followed by their destruction via the reticuloendothelial system [3, 4]. Although the development of autoantibodies by B cells remains central in the pathophysiology of ITP, a multidysfunction in cellular immunity and cytokine response may take place in the pathogenetic mechanisms of the disease [5–7].

Currently, it is generally accepted that both environmental and genetic factors are involved in the pathogenesis of ITP and, especially, interactions between genetic and epigenetic changes. Among the genetic factors, polymorphisms of inflammatory cytokine genes have been related with ITP [8–10]. In a study by Foster et al. [11], polymorphisms in Fc gamma receptors genes (*FCGR3A* and *FCGR3B*) and tumor necrosis factor-a (*TNF-a*) and lymphotoxin-a (*LTA*) genes were found to be associated with chronic childhood ITP. Satoh et al. [12] observed also an association between a polymorphism in *TNF-*β** gene and chronic ITP in adults. In addition, Wu et al. [13, 14] found that IL-4 intron 3 and IL-10 polymorphisms may contribute to childhood chronic ITP, while IL-1 Ra but not IL-1*β* exon 5 polymorphism is associated with childhood ITP.

Apart from the polymorphisms of inflammatory cytokine genes, environmentally induced epigenetic changes in gene expression have recently become a new field of research, and a lot of studies investigate the role of these changes in the loss of self-tolerance and the development of autoimmune diseases [15, 16]. Epigenetic mechanisms play an essential role in gene regulation by modifying chromatin structure, which in turn modulates gene expression. DNA methylation is a major epigenetic modification involving the addition of a methyl group to the 5′ position of a cytosine in a CpG dinucleotide and is catalyzed by DNA methyltransferases [17]. The family of DNA methyltransferases (DNMTs) consists of four independent methyltransferases, each of them playing a different functional role [18–20]. DNA methylation changes and DNMTs gene polymorphisms have been detected in several diseases, particularly cancer [21, 22]. DNMT3B promoter polymorphism has been reported to be associated with the risk of lung, colorectal, and head and neck cancers [23–26]. However, there is little information about the role of DNMTs polymorphisms in the development of autoimmune diseases [27, 28].

In the present study, we investigated the association of the rs2424913 single-nucleotide polymorphism (SNP) (C46359T) located into DNA methyltransferase 3B (*DNMT3B*) gene promoter and a VNTR polymorphism of IL-1 receptor antagonist (*IL-1 Ra*) intron-2 with an increased risk of ITP in children, in an attempt to elucidate the role of genetic and epigenetic mechanisms in the pathogenesis of such an autoimmune disease.

## 2. Patients and Methods

### 2.1. Patients and Control Subjects

The study group consisted of 32 children (17 boys and 15 girls) from unrelated families living in Crete, aged 7 months to 14 years, diagnosed with ITP, and hospitalized at the Department of Pediatric Hematology-Oncology of the University Hospital of Crete. The control group consisted of 64 individuals, sex and ethnically matched who had no history of autoimmune or other chronic diseases. The diagnosis of ITP had been made in all children based on history, physical examination, complete blood count, and examination of the peripheral smear, which should exclude other causes of thrombocytopenia. Bone marrow aspiration was performed, when necessary, to rule out other diseases. Ethnic bias within the population studied was minimized by excluding patients that were not of Cretan origin. Parents were informed that cells from the bone marrow would be used for *in vitro* research. The study had the University Hospital of Heraklion Ethics Committee approval. 

### 2.2. DNA Extraction and Analysis of the *DNMT3B* and *IL-1 Ra* Gene Polymorphisms

Whole blood was collected in EDTA-containing tubes, and genomic DNA was extracted from the peripheral blood samples using DNA purification kit: Wizard Genomic (Promega, USA) according to the manufacturer's instruction. In the study group, the genomic DNA was extracted from bone marrow mononuclear cells (BMMNCs) using the same kit. The extracted DNA was stored at −20°C to be used for the genotyping.

The subjects enrolled in this study were genotyped using the polymerase chain reaction-restriction fragment length polymorphisms (PCR-RFLP) RFLPs method. In brief, the upstream primer 5′-TGCTGTGACAGGCAGAGCAG-3′ and the downstream primer 5′-GGTAGCCGGGAACTCCACGG-3′ were used to generate a region of 380 bp of the promoter of *DNMT3B* (Table 1). The amplification was carried out by using Taq polymerase provided by Invitrogen. An initial heating step at 95°C for 5 min was used, followed by 35 cycles of denaturing (at 95°C for 30 sec), annealing (65°C for 30 sec), and chain extension (72°C for 30 sec), followed by a final extension step at 72°C for 5 min. The PCR products were digested for 3 hrs at 37°C with 5 U *Avr*II (Fermentas), which digests the DNA amplified by the T allele into two bands of 207 bp and 173 bp. In contrast, fragments carrying the major C allele lacked the *Avr*II restriction site. Both undigested and digested PCR products were analyzed through electrophoresis on 2% agarose gel and visualized (with ethidium bromide staining) under ultraviolet (UV) light in reference to a molecular weight marker.

Similarly, the upstream 5′-CTCAGCAACACTCCTAT-3′ and the downstream 5′-TCCTGGTCTGCAGGTAA-3′ primers were used to generate the IL-1 Ra region harboring the 86-bp repeats (VNTR). The amplification was performed by using 2,5 U Taq polymerase (Invitrogen). An initial denaturation step at 95°C for 5 min was used, followed by 35 cycles of denaturation (at 95°C for 30 sec), annealing (58°C for 30 sec), and chain extension (72°C for 30 sec), and a final elongation step at 72°C for 5 min. PCR products were directly analyzed by electrophoresis on 2% agarose gel and visualized upon staining with ethidium bromide. Genotypes were scored blindly, and analysis of all ambiguous samples was repeated. Moreover, 10% of the samples were amplified twice for checking the accuracy of the results. 

### 2.3. Statistical Analysis

Statistical analysis was performed using the GraphPad Prism statistical software method (GraphPad Software Inc., La Jolla, CA, USA). The distribution of the genotypes and alleles in the group of patients was compared to that of control group using the chi-squared test and Fischer's exact test where necessary, which was also used to determine whether the observed genotype frequencies conformed to Hardy-Weinberg expectations. The level of significance was set to 0.05. The association between polymorphisms and the risk of development of ITP was estimated by odds ratio (OR) and the 95% confidence intervals (CIs).

## 3. Results

### 3.1. Analysis of rs2424913 (*DNMT3B)* Polymorphism

The distribution of genotype and allele frequencies of rs2424913 *DNMT3B* SNP in 32 children with ITP and control group is presented in Table 2. Notably, no significant differences were found in the genotype distribution between the children with ITP and the control group. However, a significant difference between children with ITP and control group in allele frequencies has been observed. The frequency of allele T appeared significantly increased in children with ITP (*P *= 0.03, OR = 2, 95% CI: 1.06–3.94), thus indicating an apparent association between this allele and ITP in patients of Cretan origin. 

### 3.2. Analysis of *IL-1 Ra* VNTR Polymorphism

In case of *IL-1 Ra* polymorphism, although we found four different alleles, we focused on alleles I and II and genotypes I/I, I/II, and II/II because of their higher prevalence. The genotype and allelic distribution of IL-1 Ra among children with ITP and the control group is presented in Table 3. A statistically significant difference was observed in the allele frequencies of *IL-1 Ra* between the two groups (*P *= 0.042). Children with ITP had a significantly higher frequency of genotype I/II, compared to control group (43.75% versus 23.44%, *P* = 0.043, OR = 2.60, 95% CI: 1.02–6.50). Moreover, genotype I/I as well as allele I was overrepresented in the control group (68.75 versus 50% and 84.43 versus 71.9%), suggesting that allele I may have a decreased risk for development of ITP, whereas the presence of allele II seems to increase 2.12 times the relative risk for disease development (OR = 2.12, 95% CI: 1.02–4.41).

## 4. Discussion

Idiopathic thrombocytopenic purpura (ITP) is an autoimmune disease characterized mainly by the destruction of autoantibody-mediated platelets. Despite extensive research efforts during last years, the genetic basis of ITP remains largely unknown. In this study, by performing a case-control association study, we have investigated the possible association of the rs2424913 *DNMT3B* (C46359T) SNP and an *IL-1 Ra* VNTR polymorphism with susceptibility to ITP.

Epigenetic gene regulation has an essential role in determining individual gene function and activity. Epigenetic alterations lead to gene malfunction in a pathological context [15]. DNA methylation is a major epigenetic mechanism, which maintains chromosomal stability and regulates gene expression. It has been reported that DNA methylation plays a significant role in the development and progression of various cancers [21]. DNMT3B, analyzed in the present study, has been demonstrated to play important roles in tumorigenesis [26, 29] due to its ability to mediate de novo DNA methylation, which in turn might silence tumor suppressor gene expression through promoter hypermethylation [30]. In addition, there is an increasing interest in the role of epigenetic alterations in the pathogenesis of autoimmune diseases, such as systemic lupus erythematosus (SLE) and rheumatoid arthritis (RA) [31]. Of note, recent studies demonstrated that DNA hypomethylation has been implicated in the pathogenesis of SLE. Moreover, DNA methylation inhibitors are known to induce autoreactivity *in vitro* and the development of lupus-like syndrome *in vivo* [17, 32, 33]. There are only a few studies investigating the role of DNA methylation in the pathogenesis of ITP. Chen et al. [34] showed that there was no association between the *DNMT3B* promoter polymorphism and the susceptibility to ITP in Chinese population. However, another study reported that DNMT3A and DNMT3B mRNA expressions were significantly lower in ITP patients than in healthy controls, suggesting that aberrant DNA methylation patterns are possibly involved in the pathogenesis of ITP [35]. In the present study, we investigated the association between the rs2424913 *DNMT3B* SNP and the risk of ITP, but no significant differences were found in the genotype distribution between the children with ITP and the controls. However, we found a very low frequency of T/T genotype in our population, whereas in the Chinese population, there was found a distinct prevalence of the T/T genotype and absence of C/C genotype [34]. This finding pinpoints the importance of the racial origin in this type of studies, thus implying probably the different methylation status in different races. Moreover, we observed a significant difference in the allele distribution between children with ITP and controls. The presence of T allele seems to increase the relative risk for disease development. Altogether, our findings suggest that rs2424913 *DNMT3B* promoter SNP may be implicated to the pathogenesis of ITP. The *DNMT3B* C-to-T transition polymorphism (C46359T) examined, in *in vitro* assays, confers a 30% increase in promoter activity [23]. Although the mechanism of this association is unknown, it can be assumed that the “T” variant, by upregulating *DNMT3B* expression, may result in an aberrant *de novo* methylation of CpG islands in autoimmunity-mediating genes, thus leading to the development of ITP. However, the role of this gene in ITP seems to be a real “enigma” given that conflicting data have been presented so far. Thus, an aberrant DNA methylation status reflected by increased plasma SAH concentration and decreased expression levels of DNMT3A and 3B has been found in ITP [35], a situation that may play a crucial role in the pathophysiology of the disease. However, DNA hypomethylation (as demonstrated in the case of SLE) was found to induce autoreactivity *in vitro*. It is also possible that the “T” allele may be in linkage disequilibrium with other susceptibility loci. Altogether, the precise mechanism by which altered DNA methylation patterns induce ITP needs to be studied globally in the view of the concerted action of DNMT3A and DNMT3B, which results in a change of DNA methylation equilibrium in ITP patients.

As referred above, the pathogenesis of ITP is complicated with cellular immunity and cytokine response playing crucial roles in the pathogenesis [6, 7]. Abnormal serum cytokines levels have been reported in ITP patients [8]. The cytokine genes are polymorphic, which accounts for the different levels of cytokine production. A lot of studies have investigated so far the association between cytokine gene polymorphisms and different immunoinflammatory diseases [11, 36, 37]. IL-1 Ra, a major member of the IL-1 family (consisting of 11 members in total), is a natural anti-inflammatory molecule that neutralizes the effects of IL-1. The balance between IL-1 and IL-1 Ra is important in maintaining the homeostasis of immune system. As a result, *IL-1 Ra* polymorphisms may lead to changes in this IL-1 and IL-1 Ra balance and be associated with susceptibility of a variety of autoimmune diseases, such as rheumatoid arthritis, SLE, and ankylosing spondylitis [38–43]. There is only one study in the literature, which examined the *IL-1 Ra* polymorphism in Chinese children with ITP so far [14]. In the present study, we investigated the association between *IL-1 Ra* polymorphism and the susceptibility of ITP, and we found that *IL-1 Ra* polymorphism is associated with childhood ITP. The genotype I/II was more frequently detected in children with ITP than in controls. More specifically, we found that the presence of allele II seems to increase 2.12 times the risk for development of ITP, thus assuming that *IL-1 Ra* polymorphism may be involved in the pathogenesis of ITP. The polymorphism under investigation is caused by the variable copy number of an 86-bp sequence, and the repeat region contains three potential protein-binding sites. Therefore, the variable copy number may have functional significance. Furthermore, allele II has been reported to be associated with more severe clinical outcome in several inflammatory diseases, including systemic lupus erythematosus [41], rheumatoid arthritis, and ulcerative colitis [44]. An increased frequency of allele II has also been described in diabetes patients with nephropathy [45]. The induction of IL-1Ra by IL-1beta is an important counterregulatory mechanism and may at least partially account for the increased IL-1Ra levels found in the carriers of allele II [46, 47]. Obviously, the IL-1Ra concentrations in ITP patients can be assessed in future experiments and, if they will be found decreased, then it may suggest a deficiency of this regulatory mechanism that may be particularly pronounced in allele II carriers, thus explaining the higher incidence of ITP.

A definite advantage of our study, particularly with respect to other association studies, was the attention paid on the selection of a genetically and ethnically homogeneous patient's cohort and control group. As a consequence, the results of this study are unlikely to be biased by sampling. Given that the incidence of pediatric ITP is on the order of 4–6 cases/100,000 population annually, it is extremely difficult to collect easily more patients of Cretan origin. Crete (situated 25°E and 35°N) is the largest island of Greece, with about 0.65 million inhabitants who share the same genetic and cultural background and a common environment. A possible weakness of our study deals with the limited sample size, a fact that is difficult to be overcome easily in a geographically isolated region. 

In conclusion, our results provide evidence that rs2424913 *DNMT3B* SNP as well as the *IL-1 Ra* VNTR polymorphism may contribute to the susceptibility to ITP. However, it is in our short-term plans to collect and genotype samples from other geographical areas of Greece despite the substantial differences that may appear in the genetic background of subjects from the mainland Greece (due to increased migration or entrance of genetic material from the neighboring Balkan or west European countries in the gene pool of these Greek cohorts). In addition, further studies are needed in order to determine the functional role of the polymorphisms under study, aiming to gain insight regarding the mechanism(s) leading to ITP.

## Figures and Tables

**Table 1:** Allele types, PCR conditions, and PCR primers designed to amplify fragments harboring the polymorphic sites.

	DNMT3B promoter	IL-1 Ra
Type of polymorphism	Single-base C/T	86-bp VNTR
Site of polymorphism	Position 149	Intron 2
PCR primers		
** **Upstream	5′-TGCTGTGACAGGCAGAGCAG-3′	5′-CTCAGCAACACTCCTAT-3′
** **Downstream	5′-GGTAGCCGGGAACTCCACGG-3′	5′-TCCTGGTCTGCAGGTAA-3′
Digestion	*Avr*II	—
Allele size (bp)	C: 380	I: 410
		II: 240
	T: 207 + 173	III: 325
		IV: 500

**Table 2:** Distribution of rs2424913 *DNMT3B* allele and genotype frequencies in children with ITP and controls.

	ITP children *n* = 32 (%)	Controls *n* = 64 (%)	OR	95% CI	*P* value
*Genotype frequency*					0.07
C/C	12 (37.5)	37 (57.8)			
C/T	16 (50)	25 (39)	0.5	0.2–1.25	0.17
T/T	4 (12.5)	2 (3.2)	0.16	0.03–1.0	0.053
*Allelic frequency*					0.03*
Allele C	40 (62.5)	99 (77.34)			
Allele T	24 (37.5)	29 (22.66)	2	1.06–3.94	

^∗^ *P* < 0.05.

**Table 3:** Distribution of allele and genotype frequencies of IL-1 Ra VNTR polymorphism in children with ITP and controls.

	ITP children *n* = 32 (%)	Controls *n* = 64 (%)	OR	95% CI	*P* value
*Genotype frequency*					0.295
I/I	16 (50)	44 (68.75)	0.40^#^	0.15–0.98	
I/II	14 (43.75)	15 (23.44)	2.54^§^	1.03–6.30	0.043^∗#^
			2.60^#^	1.02–6.50	
II/II	2 (6.25)	2 (3.13)			
III/III	0 (0)	0 (0)			
I/III	0 (0)	1 (1.56)			
I/IV	0 (0)	1 (1.56)			
II/IV	0 (0)	1 (1.56)			
*Allelic frequency* ^†^					0.042*
Allele I	46 (71.9)	103 (84.43)	0.47	0.23–0.98	
Allele II	18 (28.1)	19 (15.57)	2.12	1.02–4.41	

^∗^ *P* < 0.05.

^†^Alleles from genotypes I/I, I/II, and II/II.

^#^Genotype I/I versus genotype I/II.

^§^Genotype I/II versus all genotypes.
